# Is Experience the Best Teacher? Knowledge, Perceptions, and Awareness of Wildfire Risk

**DOI:** 10.3390/ijerph18168385

**Published:** 2021-08-08

**Authors:** Giuseppina Spano, Mario Elia, Onofrio Cappelluti, Giuseppe Colangelo, Vincenzo Giannico, Marina D’Este, Raffaele Lafortezza, Giovanni Sanesi

**Affiliations:** 1 Department of Agricultural and Environmental Sciences, University of Bari Aldo Moro, Via Amendola 165/A, 70126 Bari, Italy; giuseppina.spano@uniba.it (G.S.); o.cappelluti@studenti.uniba.it (O.C.); giu.colangelo@gmail.com (G.C.); vincenzo.giannico@uniba.it (V.G.); marina.deste@uniba.it (M.D.); raffaele.lafortezza@uniba.it (R.L.); giovanni.sanesi@uniba.it (G.S.); 2 Department of Geography, The University of Hong Kong, Centennial Campus, Pokfulam Road, Hong Kong

**Keywords:** wildfire perception, exposure, wildland–urban interface, propensity score matching, questionnaire, web survey

## Abstract

Wildfires represent a natural phenomenon with detrimental effects on natural resources and human health. A better knowledge, perception, and awareness of wildfire risk may help communities at risk of exposure to prevent future events and safeguard their own lives. The aim of this study is to explore differences between individuals with and without previous wildfire experience, in terms of (1) subjective and advanced wildfire knowledge, (2) self-reported perceptions, (3) level of information, (4) self-protection measures, and (5) importance of community involvement. As a second step, we investigated differences in the same variables, focusing more deeply on a group of individuals with previous wildfire experience, classifying them according to fire-related employment (fire-related workers vs. non-workers) and wildland–urban interface (WUI) proximity (WUI residents vs. non-WUI residents). The Kruskal–Wallis test was applied to establish differences between the pairs of subsamples. Our results partially confirmed our hypothesis, that direct experience leads individuals to have a greater preparedness on the topic of wildfires. Perception of knowledge is reflected only at a shallow level of expertise, and, therefore, no relevant within-group differences related to fire-related employment or to WUI proximity were detected. Moreover, available information was perceived to be insufficient, thus we report a strong need for developing effective communication to high-risk groups, such as homeowners and fire-related workers.

## 1. Introduction

The effects of climate change coupled with unsustainable anthropic pressure are increasing wildfire risk, making European landscapes more vulnerable to wildfires [1]. Currently, the data reported by the European Commission suggest that wildfires still represent a critical issue, especially for the Mediterranean countries of Southern Europe, such as Italy. Statistics reveal that between 2009 and 2018, Italy experienced a yearly average of more than 5500 wildfire events, covering a mean annual surface area of 67,000 ha [2]. These alarming numbers reflect wildfires’ detrimental effects on natural resources and human health [3]. In this context, national and regional institutions must integrate their efforts to improve prevention, by enhancing the preparedness of communities located in areas threatened by wildfires [4]. A better knowledge, perception, and awareness of wildfire risk may help people and communities that have experienced wildfires to prevent future events and thus safeguard their own lives. 

Indeed, numerous studies have suggested that past experience of wildfires in experts, homeowners, and individuals in communities at high risk of wildfire is reflected in a greater subjective knowledge related to wildfires (e.g., risk perception, management, reduction behavior) [5,6,7,8,9]. In general, subjective knowledge related to wildfire, which can be defined as what individuals believe to know [10], has important implications for the difference in perception of risk severity, and thus in the response to that risk [8]. For instance, the behavior of homeowners has shown to be predicted by a combination of attitudes and cognitive factors (e.g., perceived behavioral control and beliefs on self-efficacy) affected by past experience with wildfires [6,7,8,9,11]. Although the relationship between past experience and subjective knowledge related to wildfire is established, mixed results are available on the relation between past experience and behavior, e.g., risk mitigation measures [12]. While some evidence states that direct experience of wildfire leads to a greater preparedness on mitigation measures [13], especially when emotional components have a crucial role, e.g., in case of property loss or evacuation [14,15], others did not confirm this relationship [11,16]. Bihari and Ryan [17] pointed out that past experience with wildfires predicts social capital and involvement in high-risk communities, which in turn affect wildfire awareness and mitigation actions.

Similarly, individuals with professional activities related to forests and fires (i.e., professionals who work directly with or manage forest areas, such as a lumberjack, forest firefighter, or forester) have shown to be more qualified in risk knowledge and perception than others, perhaps due to their prior experience [18]. However, this high-risk group has been far less investigated than wildland–urban interface (WUI) communities. 

In the face of such fragmented and inconsistent findings, it is therefore difficult to disentangle the role of direct experience in wildfire risk knowledge, perception, and awareness in individuals, whether they are homeowners, fire-related workers, or laypersons.

The overarching aim of this study is to uncover the role of direct wildfire experience in specific groups. To do this, we explored differences between individuals with and without previous wildfire experience, in terms of: (I) subjective and advanced wildfire knowledge, (II) self-reported perceptions, (III) level of information, (IV) self-protection measures, and (V) the importance of community involvement. Secondly, we investigated differences in the same variables with a greater focus on the group of individuals with previous wildfire experience, classifying them according to:

- Fire-related employment: fire-related workers vs. non-fire-related workers; and
- Wildland–urban interface (WUI) proximity: WUI residents vs. non-WUI residents.

According to the literature, we hypothesized that direct experience leads individuals to have a greater preparedness on the topic of wildfires, and that therefore no within-group differences regarding fire-related employment or WUI proximity are expected.

## 2. Materials and Methods

### 2.1. Design and study Population

This study is based on a cross-sectional design, where a single survey questionnaire was administered throughout Italy. The questionnaire was completed by 775 participants (age: mean = 37.4; standard deviation = 14.6; gender: female = 393; male = 382). The questionnaire was disseminated through various channels, including social networks and personal and professional contacts, according to a recruitment technique called “snowball sampling” or “chain-referral sampling” [19], where participants are invited to recruit other potential participants. Inclusion criteria were: (a) age ≥18 years and (b) residency in Italy. To verify that the interviewees were currently residents in Italy, they were asked to provide the municipality of residence. Geographic distribution of the sample was not homogenous; however, all the Italian regions were represented. Most of the questionnaires were received from Apulia, (southern) Sardinia, Tuscany, (central) Lazio, and Lombardy (Northern Italy).

### 2.2. Item Selection and Development

No validated questionnaire on the variables of interest is currently available in the literature. Therefore, for the item selection and development of our questionnaire we adapted previously used items for investigating the perception of wildfire risk in WUI residents in Algeria [20], Portugal [18], and the United States [6]. After merging the three aforementioned questionnaires, an adapted questionnaire resulted (see Table S1 in Supplementary Materials for the English version), suitable for the administration to a wider pool of users, including the general population, fire-related workers, and WUI residents. The adapted questionnaire consisted of six sections:

- *Sociodemographic variables and other general information*. Information on age, gender, education, occupation, and city of residence was collected. Participation in voluntary organizations or associations connected with firefighting activities was also registered, since these participants were classified as “fire-related workers”. In this section, we also asked participants to declare if they ever had a direct wildfire experience.
- *Subjective knowledge.* Variables in this section were intended to explore objective and perceived knowledge about fires, such as wildfire and WUI definitions, their occurrence, and self-perceived topic knowledge.
- *Advanced knowledge.* The four items in this section more specifically investigated those aspects related to wildfire drivers, the potential risk of an increasing number of wildfires, levels of flammability, and the role of climate change. In this section, the questions were also dichotomous.
- *Awareness of level of information.* This section explored personal beliefs about mitigation measures adopted by landscape and forest managers, and about the tendency to retrieve information on the topic. The first variable was classified as “yes” and “no”, while the second was based on a 5-point Likert scale classification, ranging from “Never” (1) to “Always” (5).
- *Self-protection measures.* This section refers to the response strategies that the participant would implement in the event of a wildfire. The two variables explored were about the response to the threat categorized as “tackling” or “avoiding the threat” (i.e., “fight or flight” response), and about the willingness to equip one’s home with fire protection systems, such as fire extinguishers and smoke detectors (“yes” and “no”).
- *Community involvement.* In this last section, we investigated the perception of the importance of scientific research, to improve the awareness of wildfire risk in the general population using a 5-point Likert scale ranging from “Not at all” (1) to “Very much” (5), and of an informed community to prevent and mitigate wildfire risk (“yes” and “no”).

Internal consistency of the total scale, as a measure of reliability, showed to be acceptable (ω = 0.7; see Dunn et al. [21] for more details).

### 2.3. Procedure

The survey was carried out in a single phase, in the period between 22 December 2020 and 22 January 2021. The amount of time required to complete the questionnaire was approximately 5 to 10 min. To administer the questionnaire, the Google Forms online platform was employed; the platform easily reaches the highest number of potential participants via several devices (e.g., tablet, smart-phone, and laptop). Moreover, COVID-19-related restrictive measures prevented face-to-face interviews.

The study procedure was designed in accordance with the ethical standards of the Helsinki Declaration and its later amendments or comparable ethical standards [22]. Potential participants were informed that participation was on a voluntary basis, the questionnaire was anonymous, and that data would be processed in an aggregate manner, in compliance with national and European data protection laws for scientific and statistical purposes (GDPR 2016/679). By choosing the option “I give my consent” all participants gave their consent to voluntarily participate in the survey and were aware that they could interrupt participation at any stage and that anonymity was guaranteed.

### 2.4. Statistical Analysis

The general survey sample was grouped into two subsamples: (a) participants with direct wildfire experience and (b) participants without direct wildfire experience. Subsequently, the subsample of participants with direct experience was further classified as follows: (c) fire-related workers vs. (d) non-fire-related workers; and (e) WUI residents vs. (f) non-WUI residents. WUI residents detected in the entire sample were 36 individuals. We paired the participants of the last two subgroups—(e) and (f)—using the propensity score matching analysis (PSM). This statistical technique allows us to balance the distributions of potentially confounding covariates, such as age, gender, and education. PSM is used to reduce selection bias by comparing groups based on covariates, and matches each participant of a sample on certain characteristics, especially in the case of statistical analyses that cannot control for such variables. [23]. Thirty-six non-WUI residents who matched for age, gender, and education with the 36 WUI residents were extracted from the total sample. In doing so, we obtained a new subsample of 36 non-WUI residents, comparable to the subsample of 36 WUI residents. PSM was performed with the package MathIt of the R Software [24]. Descriptive statistics were calculated on the six subsamples for sociodemographic variables. 

To establish differences in the responses between the pairs of subsamples, the Kruskal–Wallis test [25] was applied. Kruskal–Wallis is a nonparametric test which is used as an alternative to the analysis of variance (ANOVA) when heteroscedastic and non-normally distributed data are given [26]. The use of a nonparametric test is justified by the fact that the variables examined did not have a normal distribution, and the observations were represented by ordinal classifications. Furthermore, in the case of subsamples of WUI residents and non-WUI-residents, the sample size was too small to be able to understand if there was a normal distribution [25]. The test was performed using the statistics package of the R software.

## 3. Results

### 3.1. Descriptive Statistics

The sociodemographic characteristics of each survey subsample are displayed in Table 1. Age, gender, and education are satisfactorily balanced in each pair of subsamples. The subsample of participants with fire experience was further grouped according to the fire-related employment and WUI proximity. The descriptive statistics presented for WUI residents and non-residents refer to the matched groups after PSM analysis with the nearest-neighbor matching method, and with a ratio of one to one. Using this statistical method, we extracted from the total sample 36 non-WUI residents with a comparable age, gender, and education to the 36 WUI residents.

### 3.2. Fire Experience

As shown in Table 2, significant differences were found in the scores of the four variables related to the topic “Subjective knowledge”, namely Wildfire_definition (*x^2^* = 152.7, *p* < 0.001), Self-perceived_knowledge (*x^2^* = 17.2, *p* < 0.001), WUI_definition (*x^2^* = 189.2, *p* < 0.001), and Wildfire_occurrence (*x^2^* = 23.4, *p* < 0.001). Moreover, a significant difference in Selflearning (*x^2^* = 0.3, *p* < 0.001) was observed. The test results suggest that all the scores of the previously mentioned variables were higher in participants with fire experience than those without fire experience.

### 3.3. Fire-Related Employment

Significant differences between the subsamples of non-fire-related and fire-related workers among participants with fire experience confirmed previous findings (Table 3). “Subjective knowledge” and “Level of information” are the significant topics that yielded higher scores for fire-related than non-fire-related workers. Within them, significant differences were found for Wildfire_definition (*x^2^* = 6.2, *p* < 0.05), Self-perceived_knowledge (*x^2^* = 73.3, *p* < 0.001), WUI_definition (*x^2^* = 18.1, *p* < 0.001), Wildfire_occurrence (*x^2^* = 34.8, *p* < 0.001), and Selflearning (*x^2^* = 104.2, *p* < 0.001).

### 3.4. WUI Proximity

As a result of the Kruskal–Wallis test, three variables presented significant differences in response scores between the matched groups (Table 4). Self-perceived_knowledge within the topic “Subjective knowledge” (*x^2^* = 73.3, *p* < 0.05) and Selflearning within the topic “Level of information” (*x^2^* = 104.2, *p* < 0.001) were found to be significantly different with higher scores for WUI residents than the matched group of non-residents. On the contrary, Response_threat_categ (*x^2^* = 0.5, *p* < 0.05) showed significantly higher scores in non-WUI residents than in the WUI residents group.

## 4. Discussion

The current study investigated the differences in attitudes towards wildfires among individuals who have or have not been exposed to a wildfire. Our hypothesis was that direct wildfire experience significantly increases the perceived and actual knowledge about wildfires, their causes, mitigation measures, and need for the involvement of the social and scientific communities. Our results partially confirmed our assumptions. Individuals who have been directly exposed to a wildfire have a significantly higher level of subjective knowledge of the topic themes, e.g., definitions of “wildfire” and “wildland–urban interface”. Knowledge is also reflected by the degree of self-evaluation of the topic, which is significantly higher in the group of individuals with direct experience of a wildfire, as recent evidence supports. On the contrary, our results revealed no difference between the two groups on advanced knowledge, risk, and drivers/causes of wildfires [18].

However, we found that participants with a direct experience of wildfires requested more information on the topic than those who had never been exposed to such an event, confirming the fact that knowledge and experience influence the number of sources from which individuals usually seek information [27]. This result was confirmed in the subsamples of individuals with direct experience who have a fire-related employment, including firefighters, members of local associations, volunteers, and forest scientists. It could be inferred that these subgroups might be at risk for wildfire consequences, concerned about their own protection for work and personal reasons, and, consequently, more likely to gather information on the topic. This is in line with previous studies on both forest and wildfire specialists and WUI residents, pointing out the perceived need of further knowledge and evidence on wildfires [20,28].

Nevertheless, in our study, surprisingly no significant differences were detected among subgroups on the perception of importance of scientific research and community involvement in improving and spreading awareness of wildfire risk. In fact, previous results have revealed that forest experts consider public awareness, in particular, concerning the impact of human activities, as a crucial element for risk reduction and fire management, especially in view of the ensuing effects of climate change [29,30,31].

The perception of “mental preparedness” by experts, however, does not always translate into safe decisions [29,32]. Nevertheless, laypersons often place their trust in the knowledge and skills of experts when mitigating wildfire risk. For instance, a recent study [33] showed that not only experience with wildfires but also the perception of the effectiveness of mitigation measures shape both risk perception and the intention to implement those measures [11,34]. This is particularly true when considering homeowners, who may perceive some mitigation measures as a threat to their sense of connectedness to nature [35]. Our results showed that WUI residents, also referred to as homeowners, have a significantly higher perception of wildfire knowledge than non-WUI residents. However, this was not observed by comparing knowledge between our two subgroups. Mixed results on WUI residents’ preparedness are available in the literature, probably due to a lack of standards of measuring tools and appropriate statistical analyses [36]. Another possible explanation has been provided by Arvai et al. [37], and recently supported by Larsen et al. [38], highlighting the fact that in the phase immediately following a near-miss wildfire event there is an increase in perceived risk. At a later time, the perceived risk level naturally decreases as the individual begins to think of the event as something unlikely, and, therefore, unlikely to happen again [38]. We hypothesize that this interesting theory is a plausible explanation for the lack of difference we found in the awareness about protective measures between WUI residents and non-WUI residents. This finding is also consistent with a result in our study regarding self-protection measures. In support of previous evidence on residents’ willingness to stay and defend their properties in Victoria, Australia [39], we found that our subsample of non-residents was more willing to avoid or flee the threat of a fire, unlike WUI residents who claimed to counter it. In fact, WUI residents are almost entirely homeowners in the WUI area, with a strong place attachment to the surrounding natural environment and their communities [17,40], which makes it more difficult to leave land, homes, and other possessions to escape a fire. A possible explanation might be that WUI residents give fire protection responsibility to the government for educating residents and visitors about fire hazards and for managing public land for fire safety, whereas the responsibility is their own for fireproofing their property [9].

Lastly, an interesting result concerned the importance of an informed community and scientific research. In all subgroups, high scores were found for both dimensions as major proof of the correct perception of the usefulness of what we might define as environmental education [41].

## 5. Study Strengths and Limitations

We believe the present study is the first to have surveyed both WUI residents and fire workers, all with previous wildfire experience, throughout the Italian territory. The study, however, includes some limitations that warrant further discussion. First and foremost is the low sample number of WUI residents who have had at least one wildfire experience, and, consequently, after matching, the low number of paired non-residents. It is worth pointing out that the presence of restrictive measures resulting from the COVID-19 pandemic has made it impossible to collect data with paper and pencil tools in these areas [42]. However, considering that the WUI population in Italy is highly variable and heterogeneous [2,43], we were able to detect only a small percentage of participants recruited by word-of-mouth and social media sharing in our general sample. Another limitation is related to the use of web surveys for data collection. Despite their rapid spread as a low-cost tool for retrieving data on target populations that are difficult to reach, the reliability of web surveys depends on the level of digital education of respondents. In fact, our participant subsamples have a rather low average age, and, therefore, a predictable medium-high level of education. Such unbalanced stratification of the sample in favor of the most educated and youngest could leave aside important considerations on wildfire impact and management in at-risk communities, such as those with poor education, low income, and the elderly [44,45,46]. Future research aimed at evaluating the interplay between the characteristics of vulnerable groups and fire-related environmental variables, such as climate variability, forest fuel distribution, and topographic features is warranted [47,48]. Lastly, we acknowledge the need to use validated scales for assessing wildfire risk perception. Despite the presence of a number of scales investigating wildfire risk perception, no well-validated measurement tool is currently available. We presented an initial measure of reliability, but we hope in the future to carry out a validation and a psychometric evaluation of the proposed questionnaire.

## 6. Conclusions

Our study attempted to answer the question of whether the experience of a wildfire event is enough to acquire knowledge about wildfires. The answer is: not quite. Subjective knowledge related to wildfires is reflected only at a shallow level of expertise. In particular, contrary to our expectations, we found that the self-perceived level of knowledge of individuals with direct experience of wildfire does not translate into a real greater preparedness, except for superficial information on the subject (e.g., definitions of “wildfire” and “WUI”). Additionally, among individuals with past fire experience, not even the expected highly prepared groups (e.g., fire-related workers and WUI residents) seem to possess perceived advanced knowledge of wildfires, self-protection measures, or encouraging awareness on the importance of community involvement. However, the core of our results revealed that those groups of individuals declare to be actively engaged in seeking information on the topic, which may indicate a certain degree of self-awareness concerning the knowledge gap. It is therefore clear that the level of information of those who should provide it (e.g., the scientific community, responsible bodies) is perceived to be insufficient. Our conclusions corroborate previous studies [49,50], which highlighted the need for developing effective communication for high-risk groups, such as homeowners and fire-related workers, in order to effectively prepare them for threats and potential impacts of wildfires, and avoid the adverse health impacts of the exposure.

## Figures and Tables

**Table 1:** Descriptive statistics of the six survey subsamples.

	Fire Experience		With Fire Experience			
			Fire-Related Employment		WUI Proximity	
	With	Without	Workers	Non-Workers	Residents	Non-Residents
**Frequency (*N*)**	260	515	110	150	36	36
**Age (M ± DS)**	(38.1 ± 15.3)	(37 ± 14.3)	(37.3 ± 14.7)	(38.6 ± 15.7)	(38.7 ± 15)	(39.1 ± 15.4)
**Gender [*N* (%)]**						
Male	133 (17)	249 (32.1)	56 (22)	77 (29.6)	13 (18)	13 (18.1)
Female	127 (16)	266 (34.3)	54 (21)	73 (28.1)	23 (32)	23 (31.9)
**Education [*N* (%)]**						
No education	0	0	0	0	0	0
Primary school	0	2 (0.3)	0	0	0	0
Secondary school	25 (3.2)	27 (3.5)	8 (3.1)	17 (6.5)	6 (8.3)	6 (8.3)
High School	122 (16)	226 (29.2)	57 (22)	65 (25)	17 (24)	17 (23.6)
University or higher	113 (15)	260 (33.5)	45 (17)	68 (26.2)	13 (18)	13 (18.1)
**Occupation [*N* (%)]**						
Unemployed	15 (1.9)	23 (3)	8 (3.1)	7 (2.7)	1 (1.4)	2 (2.78)
Student	64 (8.3)	137 (17.7)	33 (13)	31 (11.9)	3 (4.2)	9 (12.5)
Tradesman	5 (0.6)	4 (0.5)	2 (0.8)	3 (1.2)	3 (4.2)	0
Employee	97 (13)	214 (27.6)	30 (12)	67 (25.8)	15 (21)	15 (20.8)
Freelancer	29 (3.7)	62 (8)	14 (5.4)	15 (5.8)	6 (8.3)	3 (4.1)
Retired	15 (1.9)	16 (2.1)	5 (1.9)	10 (3.8)	1 (1.4)	3 (4.1)
Other	35 (4.5)	59 (7.6)	18 (6.9)	17 (6.5)	7 (9.7)	4 (5.5)

N = frequency; M = mean; SD = standard deviation; WUI = wildland–urban interface.

**Table 2:** Kruskal-Wallis test for differences in the responses between participants with and without direct wildfire experience.

Topic	Item	Fire Experience						x^2^	*p* Value	
		Without (N)	With (N)	Without (M)	With (M)	Without (SD)	With (SD)			
Subjective knowledge	Wildfire_definition (Q12)	515	260	0.841	0.938	0.366	0.241	152.667	**0.000**	***
	Self-perceived_knowledge (Q13)	515	260	0.377	1.104	0.538	0.741	17.238	**0.000**	***
	WUI_definition (Q14)	515	260	0.573	0.715	0.495	0.452	189.194	**0.000**	***
	Wildfire_occurrence (Q15)	515	260	0.862	0.973	0.681	0.500	23.436	**0.000**	***
Advanced knowledge	Drivers_WUI (Q16)	515	260	0.150	0.135	0.357	0.342	72.002	0.578	
	Flammability_levels (Q17)	515	260	0.612	0.662	0.488	0.474	0.133	0.175	
	Climate_change (Q18)	515	260	0.940	0.946	0.238	0.226	2.029	0.721	
	Risk_worsening (Q19)	515	260	0.839	0.912	0.368	0.285	0.006	0.005	
Level of information	Site_specific_mit (Q20)	515	260	0.386	0.369	0.487	0.484	2.897	0.642	
	Selflearning (Q21)	515	260	2.120	3.585	1.061	1.388	0.262	**0.000**	***
Self-protection measures	Response_threat_categ (Q22)	515	260	0.728	0.696	0.445	0.461	195.801	0.350	
	Home_protection (Q23)	515	260	0.951	0.935	0.215	0.248	2.841	0.329	
Community involvement	Scientific_research (Q24)	515	260	4.200	4.258	0.940	0.934	0.312	0.361	
	Informed_community (Q25)	515	260	0.961	0.965	0.193	0.183	0.529	0.770	

Q = question; N = frequency; M = mean; SD = standard deviation; WUI = wildland–urban interface; *x*^2^ = Kruskal–Wallis chi-square; *** *p* < 0.001. Red and green shadings indicate the lower and the higher mean, respectively.

**Table 3:** Kruskal–Wallis test for differences in responses between the subsamples of non-fire-related and fire-related workers among participants with direct wildfire experience.

Topic	Item	Fire-Related Employment						x^2^	*p* Value	
		Non-Workers (N)	Workers (N)	Non-Workers (M)	Workers (M)	Non-Workers (SD)	Workers (SD)			
Subjective knowledge	Wildfire_definition (Q12)	150	110	0.907	0.982	0.292	0.134	6.182	**0.013**	*
	Self-perceived_knowledge (Q13)	150	110	0.767	1.564	0.689	0.534	73.336	**0.000**	***
	WUI_definition (Q14)	150	110	0.613	0.855	0.489	0.354	18.065	**0.000**	***
	Wildfire_occurrence (Q15)	150	110	0.960	0.990	0.196	0.095	34.849	**0.000**	***
Advanced knowledge	Drivers_WUI (Q16)	150	110	0.140	0.127	0.348	0.335	0.088	0.767	
	Flammability_levels (Q17)	150	110	0.640	0.691	0.482	0.464	0.732	0.392	
	Climate_change (Q18)	150	110	0.940	0.955	0.238	0.209	0.263	0.608	
	Risk_worsening (Q19)	150	110	0.927	0.891	0.262	0.313	1.002	0.317	
Level of information	Site_specific_mit (Q20)	150	110	0.347	0.400	0.478	0.492	0.772	0.380	
	Selflearning (Q21)	150	110	2.853	4.582	1.282	0.771	104.221	**0.000**	***
Self-protection measures	Response_threat_categ (Q22)	150	110	0.713	0.673	0.454	0.471	0.493	0.483	
	Home_protection (Q23)	150	110	0.940	0.927	0.238	0.261	0.168	0.682	
Community involvement	Scientific_research (Q24)	150	110	4.233	4.291	0.958	0.902	0.088	0.767	
	Informed_community (Q25)	150	110	0.960	0.973	0.197	0.164	0.306	0.580	

Q = question; N = frequency; M = mean; SD = standard deviation; WUI = wildland–urban interface; *x*^2^ = Kruskal–Wallis chi-square; * *p* < 0.05; *** *p* < 0.001. Red and green shadings indicate the lower and the higher mean, respectively.

**Table 4:** Kruskal–Wallis test for differences in responses between the subsamples of non-WUI and WUI residents among participants with direct wildfire experience.

Topic	Item	WUI Proximity						x^2^	*p* Value	
		Non-Resident (N)	Resident (N)	Non-Resident (M)	Resident (M)	Non-Resident (SD)	Resident (SD)			
Subjective knowledge	Wildfire_definition (Q12)	36	36	0.889	0.972	0.319	0.167	6.182	0.167	
	Self-perceived_knowledge (Q13)	36	36	0.917	1.361	0.806	0.593	73.336	**0.015**	*
	WUI_definition (Q14)	36	36	0.694	0.833	0.467	0.378	18.065	0.168	
	Wildfire_occurrence (Q15)	36	36	0.972	0.944	0.166	0.232	34.849	0.336	
Advanced knowledge	Drivers_WUI (Q16)	36	36	0.083	0.111	0.280	0.319	0.088	0.693	
	Flammability_levels (Q17)	36	36	0.694	0.611	0.467	0.494	0.732	0.461	
	Climate_change (Q18)	36	36	0.917	1.000	0.280	0.000	0.263	0.079	
	Risk_worsening (Q19)	36	36	0.944	0.944	0.232	0.232	1.002	1.000	
Level of information	Site_specific_mit (Q20)	36	36	0.333	0.361	0.478	0.487	0.772	0.806	
	Selflearning (Q21)	36	36	3.111	4.167	1.389	1.134	104.22	**0.001**	**
Self-protection measures	Response_threat_categ (Q22)	36	36	0.639	0.389	0.487	0.494	0.493	**0.035**	*
	Home_protection (Q23)	36	36	0.861	0.917	0.351	0.280	0.168	0.456	
Community involvement	Scientific_research (Q24)	36	36	4.111	4.389	1.008	0.803	0.088	0.261	
	Informed_community (Q25)	36	36	1.000	1.000	0.000	0.000	0.306	NA	

Q = question; N = frequency; M = mean; SD = standard deviation; WUI = wildland–urban interface; NA = not applicable; x^2^ = Kruskal–Wallis chi-square; * *p* < 0.05; ** *p* < 0.01. Red and green shadings indicate the lower and the higher mean, respectively.

## Data Availability

The study did not report any data.

## Supplementary Materials

The following are available online at https://www.mdpi.com/article/10.3390/ijerph18168385/s1, Table S1: survey questionnaire (English translation).
