# Supplementary material for: Is Experience the Best Teacher? Knowledge, Perceptions, and Awareness of Wildfire Risk
Source: Int J Environ Res Public Health. 2021 Aug 8;18(16):8385. doi: 10.3390/ijerph18168385 (PMC8394653; doi:10.3390/ijerph18168385)
Supplement: Supplementary file 1 [file ijerph-18-08385-s001.zip › ijerph-1290431-supplementary.pdf]

Supplementary Material

Survey questionnaire (English translation)

"Knowledge, Perception, and awareness of wildfire risk"

Date………

A. ABOUT YOU

1. Age: _ _ years old
2. Gender:
   - o Male
   - o Female
   - o Third Gender
3. Education level:
   - o No education
   - o Primary school
   - o Secondary school
   - o High school
   - o University or higher
4. Occupation:
   - o Unemployed
   - o Student
   - o Tradesman
   - o Employee
   - o Freelancer
   - o Retired
   - o If other, please specify: ________________________________
5. Nationality: __________________________
6. City of residence: _______________________
7. Do you work or volunteer for organizations or associations (e.g., universities, civil protection, fire fighters) that are connected or that deal with fires?
   - o Yes
   - o No
8. If yes, please specify which: ______________________
9. In which area of your municipality do you live?
   - o Urban, peri-urban, or rural area
   - o Forest area
10. If you have a second home, in which area of the municipality is it located?
    - o Urban, peri-urban, or rural area
    - o Forest area
11. Have you ever experienced a forest fire firsthand?
    - o Yes
    - o No

B. SUBJECTIVE KNOWLEDGE

12. How do you define a “wildfire”?

- As burning of the stubble, straw, and vegetation present at the end of the production cycles of natural meadows or arable land
- As the combustion of artifacts, infrastructures, and buildings in the vicinity of wooded areas
- As a fire capable of spreading over wooded, bushy, or arable land

13. How do you rate your knowledge about wildfires?

- No or little knowledge
- Moderate knowledge
- High knowledge

14. How do you define a “Wildland-Urban Interface”?

- A graphical interface that allows you to manage and map urban and forest areas
- Areas where homes and infrastructures meet and mix with wooded or natural areas
- Areas where housing and infrastructure meet and mix with rural or agricultural areas

15. How often do wildfires occur in your region?

- Never
- At least once a year

C. ADVANCED KNOWLEDGE

16. Do you think that housing located in forest areas are protected from wildfires?

- Yes
- No

17. Please select which vegetation seems to you the most flammable (select the corresponding letter):

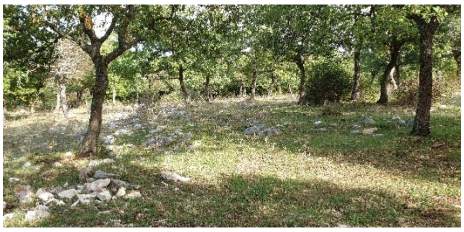
(a)

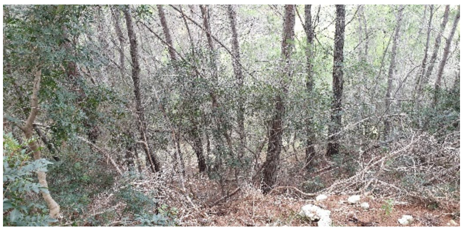
(b)

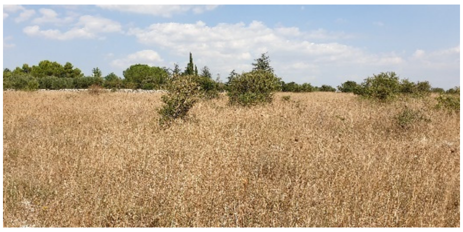
(c)

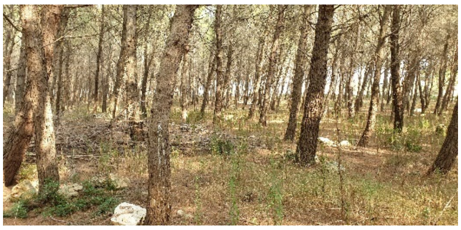
(d)

18. Do you think climate change can affect wildfire risk?

- Yes
- No

19. Do you think the wildfire risk in your region may increase?
    - o Yes
    - o No

D. AWARENESS OF LEVEL OF INFORMATION

20. Do you think that sufficient measures have been applied to mitigate the wildfire risk in your region?
    - o Yes
    - o No
21. Do you look for information about wildfires?
    - o Never
    - o Rarely
    - o Sometimes
    - o Often
    - o Always

E. SELF-PROTECTION MEASURES

Please, assume you are in a wildfire risk area…

22. …what would you do?
    - o I would counter the threat
    - o I would avoid the threat
23. …would you equip your home with fire protection systems?
    - o Yes
    - o No

F. COMMUNITY INVOLVEMENT

24. Do you think that scientific research is essential to improve people's level of awareness of wildfire risk?
    - o Not at all
    - o A little
    - o Neutral
    - o Somewhat
    - o Very much
25. Do you think that a more informed and prepared community is an important condition for the prevention and mitigation of wildfire risk?
    - o Yes
    - o No
